# Effect of the Dysbindin Gene on Antimanic Agents in Patients with Bipolar I Disorder

**DOI:** 10.4306/pi.2008.5.2.102

**Published:** 2008-06-30

**Authors:** Dong-Hwan Yun, Chi-Un Pae, Antonio Drago, Laura Mandelli, Diana De Ronchi, Ashwin A. Patkar, In Ho Paik, Alessandro Serretti, Jung-Jin Kim

**Affiliations:** 1 Department of Psychiatry, Kangnam St. Mary's Hospital, The Catholic University of Korea College of Medicine, Seoul, Korea.; 2 Department of Psychiatry and Behavioral Sciences, Duke University Medical Center, Durham, NC, USA.; 3 Institute of Psychiatry, University of Bologna, Bologna, Italy.

**Keywords:** Pharmacogenetics, Dysbindin, Bipolar disorder, Antimanic agents

## Abstract

**Objective:**

We previously reported an association between dysbindin gene (DTNBP1) variants and bipolar I disorder (BID). This paper expands upon previous findings suggesting that DTNBP1 variants may play a role in the response to acute mood stabilizer treatment.

**Methods:**

A total of 45 BID patients were treated with antimanic agents (lithium, valproate, or carbamazepine) for an average of 36.52 (±19.87) days. After treatment, the patients were evaluated using the Clinical Global Impression (CGI) scale and the Young Mania Rating Scale (YMRS) and genotyped for their DTNBP1 variants (rs3213207 A/G, rs1011313 C/T, rs2005976 G/A, rs760761 C/T and rs2619522 A/C).

**Results:**

There was no association between the variants investigated and response to mood stabilizer treatment, even after considering possible stratification factors.

**Conclusion:**

Although the small number of subjects is an important limitation in our study, DTNBP1 does not seem to be involved in acute antimanic efficacy.

## Introduction

Bipolar disorder is known to be affected by genetic factors.^1^-^3^ Although it is not a classical neurodegenerative disease, bipolar disorder has been recently reported to be associated with a neurodegenerative pathophysiology.^4^ Moreover, cell-life promoting events are reasonably responsible for neuronal plasticity and resilience, which have been recognized as relevant factors in the development of bipolar disorder.^5^ Therefore, the dysbindin seems to be a good candidate as it is associated with both intracellular trafficking and signal transduction.^6^,^7^

Lithium, valproate and carbamazepine have been considered as the first line treatments for both the acute and long-term treatment of patients with bipolar disorder.^8^ Given the paucity of the clinical predictors of response,^9^ pharmacogenetic predictors would be of great assistance to clinicians. The mood-stabilizing, prophylactic, and neuroprotective effects of lithium in association with variations of numerous genes have been reported in genome-wide studies.^10^-^12^

However, carbamazepine and valproate have not been as widely investigated as lithium. Nevertheless, their prophylactic activities have been associated with some genes or proteins,^13^,^14^ providing some rationale for a pharmacogenetic approach to investigating their acute antimanic effects. This field of research is particularly difficult and intriguing since the specific mechanism of action leading to mood stability has yet to be identified^14^,^15^ and because the genetic inheritance of acute antimanic response remains unclear. Even though the prophylactic action of mood stabilizers has been widely investigated, pharmacogenetic studies investigating the acute antimanic effect of mood stabilizers are lacking.

Therefore, our group used a pharmacogenetic approach to investigate the possibility that dysbindin gene has any effects on response to antimanic agents. A portion of the coding sequence for dysbindin gene (rs3213207; rs1011313; rs760761; rs16876759; rs2619522) was chosen on the basis of evidence of an association between dysbindin and depressive spectrum disorders, bipolar disorder and schizophrenia.^16^-^19^ Genetic investigations were performed on Korean patients with bipolar disorder.

## Methods

### Subjects

The subjects were 45 inpatients (20 males) who had been diagnosed with Bipolar I Disorder (BID) and scored at least 13 on the Young Mania Rating Scale (YMRS) at baseline. The clinical diagnosis was confirmed by the consensus of two board-certified psychiatrists based on Diagnostic and Statistical Manual of Mental Disorders IV (DSM-IV) criteria,^20^ and patients with comorbid Axis I disorders other than BID were excluded. Subjects with neurological and current medico-surgical illnesses were also excluded. Patients were administered the Clinical Global Impression (CGI) scale and the YMRS at admission. The patients were assessed for the retest sessions just before their discharge following the cessation of the manic symptoms.

All subjects were biologically unrelated, native Korean descendants currently residing in Korea. Written informed consent was provided by the subjects after the purpose and method of the study had been explained. The institutional review board of The Catholic University of Korea, Kangnam St. Mary's Hospital approved the study, which was conducted in accordance with the Declaration of Helsinki.

### Genetic analyses

The dysbindin gene, DTNBP1, was initially cloned by Benson and colleagues.^6^ It is located at position 6p 24-2 and has three known isoforms: A, B and C. Isoforms A and C have 10 exons spanning approximately 140 kb, while isoform B has 10 exons spanning approximately 139 kb.

Genomic DNA was extracted from blood by standard methods and then quantified. The high-throughput genotyping method using pyrosequencer (Biotage AB, Sweden) was used to genotype 5 single nucleotide polymorphisms (SNPs) (rs3213207 A/G, rs1011313 C/T, rs2005976 G/A, rs760761 C/T and rs2619522 A/C) of the dysbindin gene. Genetic variants were selected from a public database (National Center for Biotechnology Information, dbSNP, http://www.ncbi.nlm.nih.gov/SNP/).

Polymerase chain reaction (PCR) primers (Bioneer, Daejeon, Korea) and sequencing primers (Bioneer, Daejeon, Korea) used for the pyrosequencing assay were designed using the Pyrosequencing Assay Design Software v1 (Biotage AB, Sweden), and one primer from each primer set was biotinylated.

### Statistical analyses

Haploview 3.2 was used to generate a linkage disequilibrium map and to test for Hardy-Weinberg equilibrium. Single genotype associations with YMRS and CGI scores were analyzed by analysis of variance (ANOVA). When including covariates or other factors, analysis of covariance (ANCOVA) and multivariate analysis of co-/variance (MANOVA/MANCOVA) were employed. Baseline scores were included as covariates in addition to the clinical variables associated with genotypes. Comparisons with other clinical variables in the sample were performed using ANOVA or the Chi-square test. The "R" software ("A Programming Environment for Data Analysis and Graphics" Version 2.2.1) was used to analyze the haplotypes with both discrete and continuous traits and to include covariates. Permutation (50,000 permutations) was used to estimate the global significance of the results of the haplotype analyses in order to confirm the expectation-maximization values. Results were considered significant with an alpha level lower than 0.05.

With this level of significance (alpha=0.05), for single marker allele analyses, we had a power of 0.80 to detect a medium-large effect size of d=0.97, which corresponded to a difference of approximately 4.5% in YMRS improvement between the two main genotype variants and corresponded to an explained variance of about 19.6%.

## Results

The clinical characteristics of the patients are presented in Table 1. A significant reduction in YMRS mean score was reported (64.20±4.55) for the whole sample. Subjects treated with lithium, valproate and carbamazepine had 64.94 (±4.71), 64.15 (±2.84) and 60.71 (±5.83) reductions in YMRS scores, respectively. The present study was not designed to investigate the different efficacies of the single drugs, so we did not perform any statistical investigation in that direction.

In the clinical correlation analysis, we observed a significant positive correlation (R Spearman=0.63, p<0.0001) between YMRS scores at baseline and the % reduction in YMRS score by the end of treatment. We also observed an inverse association with age at onset and the % reduction in YMRS score (R Spearman=-0.29, p=0.05). When considering the dichotomic variable, the results of the "remitters" and "non remitters" were similar.

In regards to the genetic analysis of the dysbindin sequence, all markers were in Hardy-Weinberg equilibrium (rs3213207 p=0.94; rs1011313 p=0.55; rs760761 p=0.44, rs16876759 p=0.44, rs2619522 p=0.39).

The DTNBP1 variations were not associated with the clinical variables of the sample or with the acute antimanic effects of lithium, valproate and carbamazepine. In addition, no association was observed when possible stratification factors were included in the analysis (age at onset, kind of drug, period of administration, administered doses, YMRS at baseline, CGI at baseline). Haplotype analyses showed no significant association with the effect of acute antimanic treatment. Haplotypes were not associated with baseline YMRS (global p=0.78). Analysis of covariance for age and baseline scores also showed that the haplotypes were not associated with final YMRS (global p=0.57) scores.

## Discussion

In the present study, we did not find an association between the variations within the investigated DTNBP1 coding sequence variants and the acute antimanic effects of lithium, valproate and carbamazepine. Minor clinically significant results were obtained: there was a significant positive correlation between baseline YMRS score and YMRS % score reduction by the end of treatment. We also observed an inverse association between age at onset and reduction in YMRS % score.

Nevertheless, DTNBP1 can still be considered as a promising candidate for acute antimanic effects, as there is some evidence that dysbindin modulation in the glutamatergic and GABAergic systems is related to the neurotransmitter imbalance associated with acute manic symptoms,^5^,^15^,^21^ and that some antiglutamatergic agents, such as riluzole and lamotrigine, have antidepressant effects.^22^

Moreover, it has been suggested that drugs that reduce glutamatergic activity or glutamate receptor-related signal transduction may also have antimanic effects.^23^ Finally, GABAergic tone has been associated with bipolar disorder and its treatment.^24^ DTNBP1 variants have been consistently associated with bipolar disorder,^25^ and we reported that some DTNBP1 variants are significantly associated with the response of antidepressants.^18^ In contrast, Raybould et al.^26^ reported that dysbindin gene variants were not associated with bipolar disorder, and Zill et al.^27^ reported that variations in the DTNBP1 gene had no effect on the etiology of major depression or on the response to antidepressant treatment, which is in agreement with the finding of the present study. Nonetheless, further investigation into the DTNBP1 gene is warranted.

The negative result presented in this paper may have resulted from several limitations, including the small sample size, heterogeneity, various durations of treatment and different doses of drugs. Drug plasma level might be an important missing variable. The lack of genomic control to exclude stratification bias may be another limitation of the study; however, the Korean population is considered to be genetically homogenous. Considering the short duration of treatment with antimanic agents in this study (36.5±19.9), the association between the DTNBP1 gene and the effects of antimanic drugs on the long-term treatment of bipolar I disorder should be confirmed.

Finally, we investigated only a portion of the DTNBP1 coding sequence, on the basis of evidence in the abovementioned literature.

In conclusion, there was no association between genetic variations in the DTNBP1 coding sequence and the acute antimanic effects of lithium, valproate and carbamazepine in the present study. Further research with a larger sample is needed to confirm the role of DTNBP1 in the effects of antimanic drugs in patients with BID.

## Figures and Tables

**TABLE 1:**
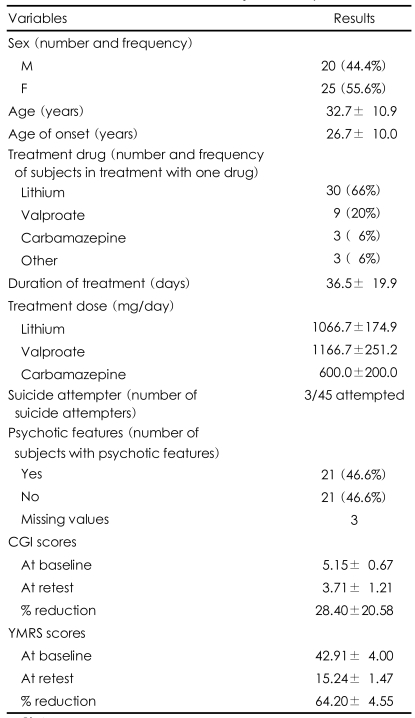
Clinical characteristics of subjects with bipolar I disorder

CGI: Clinical Global Impression, YMRS: Young Mania Rating Scale
